# Laser-pointer-induced self-focusing effect in hybrid-aligned dye-doped liquid crystals

**DOI:** 10.1038/srep09890

**Published:** 2015-05-06

**Authors:** Jing Wang, Yosuke Aihara, Motoi Kinoshita, Jun-ichi Mamiya, Arri Priimagi, Atsushi Shishido

**Affiliations:** 1 Chemical Resources Laboratory, Tokyo Institute of Technology, R1-12, 4259 Nagatsuta, Midori-ku, Yokohama 226-8503, Japan; 2 Department of Chemistry and Bioengineering, Tampere University of Technology, Korkeakoulunkatu 8, Tampere, 33720, Finland; 3 PRESTO, JST, 4-1-8 Honcho, Kawaguchi 332-0012, Japan

## Abstract

Nonlinear optics deals with phenomena where “light controls light”; e.g., there is mediation by an intensity-dependent medium through which light propagates. This field has attracted much attention for its immense potential in applications dependent on nonlinear processes, such as frequency conversion, multiple-photon absorption, self-phase modulation, and so on. However, such nonlinearities are typically only observed at very high light intensities and thus they require costly lasers. Here, we report on a self-focusing effect induced with a 1 mW handheld laser pointer. We prepared polymer-stabilized dye-doped liquid crystals, in which the molecular director orientation gradually changes from homeotropic at one surface to homogeneous at the other. This is referred to as hybrid alignment. In such films, the threshold intensity needed to form diffraction rings was reduced by a factor of 8.5 compared to that in conventional homeotropic cells, which enabled the induction of the self-focusing effect with a laser pointer.

Nonlinear optics (NLO) describes the behaviour of light in nonlinear media, for which the dielectric polarization responds to the incoming light field in a nonlinear manner^1^,^2^. NLO has immense potential in applications dependent on nonlinear processes, for instance, frequency conversion^3^,^4^, multiple-photon absorption^5^,^6^, and self-phase modulation^7^,^8^. However, the generation of such optical nonlinearities typically requires very high light intensities and thus costly lasers. Crystals^9^, polymers^3^,^10^, and liquid crystals (LCs)^11^ exhibiting high optical nonlinearity have been extensively studied with the aim of creating NLO effects using inexpensive, low-power lasers. The intensity-dependent refractive index, one of the useful NLO effects, evokes self-focusing, self-phase modulation, phase conjugation, and the generation of optical solitons. It thereby possesses great potential for use in a range of photonic applications, such as optical switching, data storage, and light-beam modulation^12^,^13^,^14^,^15^.

In 1980s, LCs became firmly established as highly efficient NLO materials, due to the ease of their light-induced molecular director orientation and the resultant huge change in their refractive index^16^. Later, this orientational optical nonlinearity was further enhanced by adding a small amount of absorbing dye molecules to the liquid crystal^17^,^18^. Theoretical and experimental studies of both the molecular arrangement and the dye structure were reported. In 2004, it was reported that the change in refractive index caused by molecular reorientation could be induced even using light intensities in the range of nW/cm^2^ in azobenzene-doped LCs in a glass cell with no surface treatment^19^. With this report, researchers reached a final consensus on the NLO material with the highest nonlinearity. This resulted in the slowing of further exploration from the viewpoint of low-intensity-induced refractive index change. However, these colossal optical nonlinearities were elicited and measured with a holographic grating setup, which restricted the practical use of optical nonlinearity due to its requirement for two-beam interference. Additionally, non-treated LC cells exhibit huge optical scattering, which is also a problem for photonic applications. In contrast, self-focusing is simply triggered by single beam irradiation and is expected to have a wide variety of applications^11^. Nevertheless, low-intensity-driven self-focusing materials remained unexplored. We have recently found that oligothiophene (TR5)-doped LCs stabilized by photopolymerization exhibit an unexpectedly low threshold intensity for self-focusing^20^. The occurrence of self-focusing at even lower light intensities would allow for its flexible and broad-ranging use in photonic applications.

Here, we report that the light intensity needed to induce the self-focusing effect can be reduced to levels accessible with a common 1 mW handheld laser pointer in polymer-stabilized TR5-doped LC (PSLC). The key to this achievement is the use of hybrid molecular alignment instead of the commonly used homeotropic or planar LC alignment. Hybrid alignment refers to homeotropic (out-of-plane) alignment at one substrate of the LC cell, while homogeneous (in-plane) alignment occurs at the other substrate, as illustrated in Fig. 1a. Hybrid alignment has been investigated mostly from a theoretical perspective for issues such as LC molecular alignment^22^ and light propagation behaviour^23^. We chose to use hybrid LC alignment because of the unique properties of hybrid-aligned cells. These include optical transparency, a bistable surface, and opposed surface anchoring, which might enhance reorientational optical nonlinearities in LC systems. The NLO response of the hybrid-aligned TR5-doped PSLC film was compared to homeotropic-aligned film with the same composition. Both of those surfaces were treated with a silane coupler (Fig. 1b).

## Results

### Characterization

**Figure 1:**
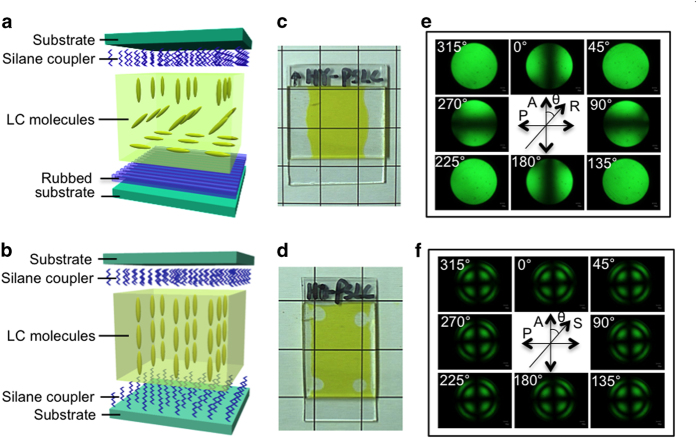
Geometry and characterization. (**a**) Sketch of the geometry of a hybrid-aligned cell. (**b**) Sketch of the geometry of a homeotropic-aligned cell. (**c**) Photograph of a hybrid-aligned cell. (**d**) Photograph of a homeotropic-aligned cell. (**e**) Polarized optical micrograph of a hybrid-aligned cell on a rotatable stage. (**f**) Polarized optical micrograph of a homeotropic-aligned cell on a rotatable stage. Optically transparent film with hybrid alignment is confirmed from photograph and conoscopic images with a polarized optical microscope.

The fabrication details of hybrid-aligned and homeotropic-aligned TR5-doped PSLC are described in the Methods section. Both cells were transparent and of high optical quality (Fig. 1c and 1d), and the hybrid and homeotropic molecular alignment was confirmed by polarized optical microscopy (Fig. 1e and 1f).

### Evaluation of the self-focusing effect

**Figure 2:**
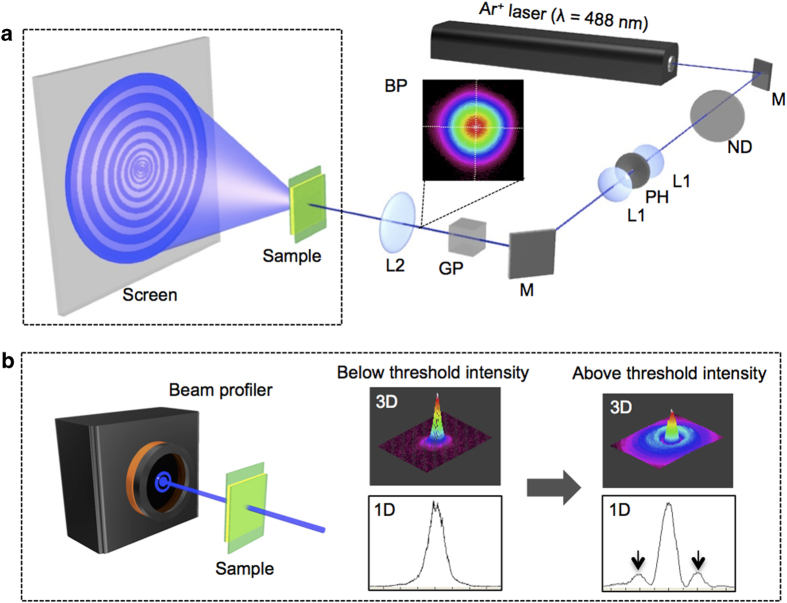
Optical setup for self-diffraction ring measurement. (**a**) Schematic diagram of the optical setup. The beam diameter before L2 was 2 mm, and the sample was placed at the focal point of L2 upon normal incidence where the beam size was 50 μm. M1, mirror; ND, neutral density filter; L1, plane convex lens; PH, pinhole; GP, Glan-Thompson prism (set to vertical polarization); L2, biconvex lens (f = 15 cm); BP, beam profile. (**b**) Illustration of the threshold light intensity determination using a beam profiler (left) by observation of the 3D and 1D images of the beam (right).

**Figure 3:**
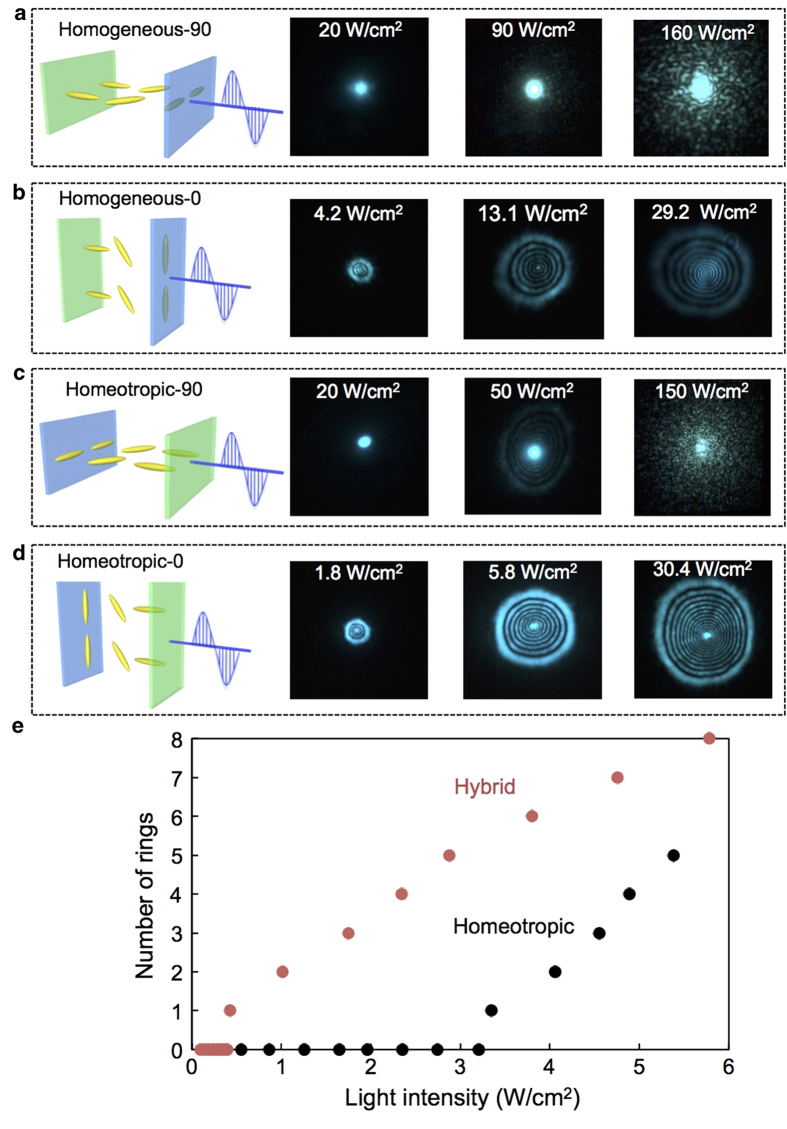
Nonlinear optical response in a hybrid-aligned cell and comparison with a homeotropic-aligned cell. (**a**–**d**) Sketch of the arrangement (left) and self-diffraction ring observation at three light intensities (right). The homogeneous or homeotropic label on the sketch indicates which surface is facing the incident light, and 0 or 90 indicates the angle between the rubbing direction and the laser polarization. (**a**) Homogeneous-90. (**b**) Homogeneous-0. (**c**) Homeotropic-90. (**d**) Homeotropic-0. (**e**) Number of diffraction rings as a function of light intensity for the hybrid-aligned cell and the homeotropic-aligned cell. The first ring appears only at 0.4 W/cm^2^ in the hybrid-aligned cell, indicating a reduction in the threshold by a factor of 8.5.

The threshold intensity to show self-focusing effect was evaluated by self-diffraction ring measurements. A spatially filtered, vertically polarized Ar^+^ laser beam (488 nm) with a Gaussian intensity distribution was focused onto the sample cell at normal incidence (Fig. 2a). The diffraction ring pattern due to self-phase modulation was observed on a screen placed behind the sample, and the number of rings was used to indicate the refractive index modulation at different light intensities^24^. The threshold intensity, at which the first diffraction ring formed, was determined with a beam profiler (Fig. 2b). Due to the complicated alignment of hybrid-aligned LC cells, we carefully studied the diffraction ring formation for four different cell placement arrangements (Fig. 3), depending on the surface facing the incident light and the angle between the rubbing direction and the polarization direction of the incident laser beam: (1) homogeneous-90, (2) homogeneous-0, (3) homeotropic-90, and (4) homeotropic-0. The polarized UV-Vis spectra at those four cell arrangements are shown in Supplementary Fig. S3a.

Figure 3 shows the nonlinear optical reorientation in hybrid-aligned TR5-doped PSLC. When the light was incident to the sample from the surface with homogeneous LC alignment, no rings were observed when the laser polarization was perpendicular to director orientation (homogeneous-90; Fig. 3a). After we rotated the cell so that the polarization direction coincided with the molecular director (homogeneous-0), then diffraction rings appeared above a threshold intensity of 1.4 W/cm^2^, and their number increased with increasing light intensity (Fig. 3b). Upon irradiation of the cell from the homeotropic side, a few rings were observed at >50 W/cm^2^ intensity when the laser polarization was perpendicular to director orientation; furthermore, at overly high intensities, a thermal disorder pattern occurred (Fig. 3c). Finally, when the cell was rotated by 90° (homeotropic-0), a clear concentric ring pattern appeared at very low intensities as demonstrated in Fig. 3d. The maximum number of rings achieved with this geometry was 14.

The diffraction rings can be generated by either an order-to-order molecular reorientation or an order-to-disorder photothermal change in refractive index. The former is usually preferred because it allows more precise control over the molecular alignment and hence over the light propagation through the cell. To determine the origin of the rings, we performed a pump probe experiment (Supplementary Fig. S1a) and observed the polarization dependence of the ring pattern with a non-resonant He-Ne probe beam. For homeotropic-90, the rings exhibited no light polarization dependence (Supplementary Fig. S1c), suggesting a photothermal effect. On the other hand, the rings for the homogeneous-0 and homeotropic-0 arrangements exhibited clear polarization dependence (Supplementary Fig. S1b and Fig. S1d); hence, in these cases the ring formation can be attributed to photoinduced order-to-order molecular reorientation. Different light pathways through the cell and the resulting different light absorbances probably determine the numbers of rings in the two arrangements.

The self-focusing effect in the hybrid-aligned cells was confirmed to be reversible, which is another important feature affecting potential applications. We studied the reversibility by repeatedly measuring the light from a He-Ne probe beam transmitted through the centre of the diffraction rings upon five subsequent on-off cycles with 200 s intervals (Supplementary Fig. S2a). Identical transmittance changes were observed over several cycles (Supplementary Fig. S2b), which proved that the nonlinear optical molecular reorientation in the hybrid-aligned cell was reversible. It must be noted that the optical response in the hybrid-aligned cell was accelerated by a factor of 10 compared to a conventional homeotropic-aligned cell (Supplementary Fig. S2c).

The threshold intensity of the hybrid-aligned PSLC cell was at its lowest (0.4 W/cm^2^) in the homeotropic-0 arrangement, that is, when light having polarization parallel to the rubbing direction was incident on the sample from the homeotropic side. The threshold was then compared to that of the homeotropic-aligned PSLC sample with the same composition. As shown in Fig. 3e, the threshold intensity was markedly reduced in the hybrid-aligned cell by a factor of 8.5 compared with that of homeotropic-aligned PSLC (3.4 W/cm^2^). According to Supplementary Fig. S3, the absorbance at 488 nm was much higher for hybrid-aligned PSLC (0.45) compared with that of homeotropic-aligned PSLC with the same composition (0.14). Hence, we also compared the threshold of hybrid-aligned PSLC with that of the homeotropic-aligned PSLC with the same absorbance (0.44, with 0.32 mol% of TR5). The threshold intensity for homeotropic-aligned PSLC (0.32 mol% TR5) was 2.3 W/cm^2^, which indicates that the threshold was also reduced in hybrid-aligned PSLC by a factor of 5.8 even when compared with homeotropic-aligned PSLC with the same absorbance.

### Laser-pointer-induced self-focusing effect

**Figure 4:**
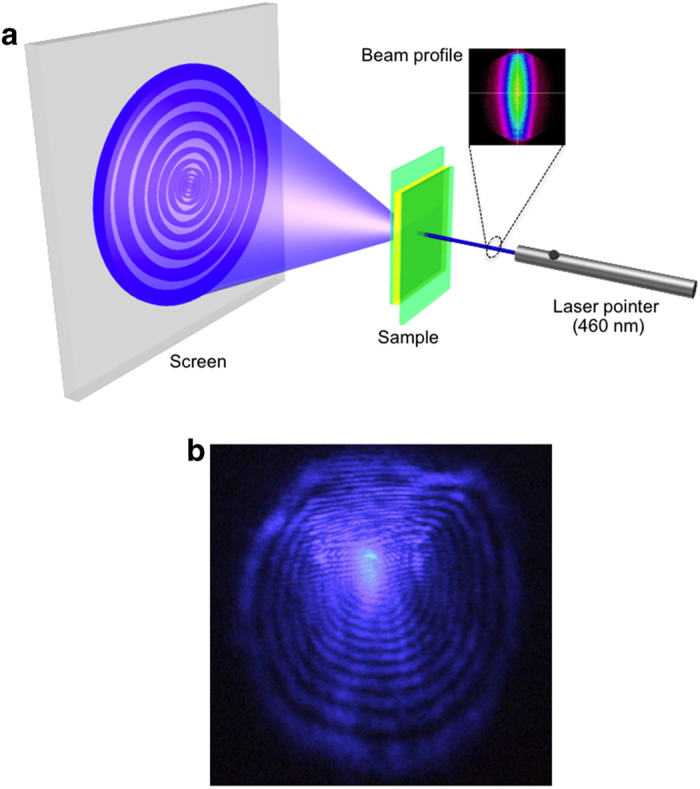
Laser-pointer-driven nonlinear optical effect. (**a**) Sketch of the optical setup for the observation of the laser-pointer-driven nonlinear optical effect in a hybrid-aligned cell. (**b**) Typical laser-pointer-driven ring pattern generated from a hybrid-aligned cell (TR5: 0.03 mol%). A 1 mW commercially available laser pointer drives molecular reorientation, leading to self-focusing and self-phase modulation.

As discussed above, hybrid molecular alignment markedly decreases the threshold intensity for self-focusing to 0.4 W/cm^2^. To the best of our knowledge, this is the lowest value obtained for the order-to-order type of self-focusing effect (from the original molecular alignment to the molecular director parallel to light polarization). Hence, the laser power required for nonlinear optical molecular reorientation could be in the sub-100 μW range and would be accessible with common low-power handheld laser pointers. Therefore, we explored driving this nonlinear optical effect using an extremely simple setup as sketched in Fig. 4a. The laser polarization was horizontal while the cell was set as a homeotropic-0 arrangement. A 1 mW battery-operated laser pointer (460 nm, Shenzhen Keyuan Co., Ltd., China) with a small lens was employed. Considering the much higher absorbance at 460 nm in the hybrid-aligned cell, we decreased the dye concentration of TR5 to 0.03 mol% (Supplementary Fig. S3d). As shown in Fig. 4b, the diffraction ring patterns due to self-focusing and self-phase modulation immediately appeared in the hybrid-aligned cell merely by turning on the laser pointer (Supplementary Movie S1).

We would like to emphasize that the nonlinear optical effect is incident-direction sensitive because of the asymmetric molecular arrangement in the hybrid cell (Supplementary Movie S2); when the beam was incident from the homogeneous side, rings were not formed. However, by just rotating the film so that the beam was incident from the homeotropic side, the rings became immediately observable. This means that the cell functions as the film type “nonlinear optical diode”, which exhibits the desired optical response only above a certain light intensity. A film that has light transmission ability on one side simultaneously with protection ability on the other side might be used on integrated optical devices with microscopically arranged light emitters and photodetectors.

## Discussion

Why does the hybrid alignment decrease the threshold intensity so markedly? Although the initial molecular states in hybrid-aligned and homeotropic-aligned cells are different, their final molecular alignment states are the same; that is, the molecules reorient to be parallel to the incident light polarization. The surface anchoring direction in the homeotropic-aligned cell is perpendicular to the final molecular alignment. Therefore, the molecules experience a strong torque that tends to align them perpendicularly to the surface. On the other hand, for a hybrid-aligned cell, the surface anchoring direction at the rubbed surface is parallel to the final molecular alignment direction, and the torque originates from the homeotropic surface only. Consequently, the torque needed to align the molecules back to the initial state during photoirradiation is reduced in hybrid-aligned cells, which both decreases the reorientation threshold intensity and accelerates the molecular realignment compared to homeotropic cells.

The materials proposed here are highly flexible as far as optimizing the optical nonlinearity. Unlike in crystalline NLO materials, the chemical structure of both the dopant and the host can be individually tailored, and the performance of the dopant concentration, film thickness, and polymerization conditions can be optimized. Furthermore, large-area films can be fabricated using display technology. By further reducing the threshold light intensity of the self-focusing effect, this effect can be applied to more common light sensitive materials, such as those in displays, optical-limiting windows, protective goggles, and so on.

In summary, we demonstrated the nonlinear optical effect of hybrid-aligned olighothiophene-doped polymer-stabilized LC film. Self-focusing was enabled by photoinduced molecular reorientation at the lowest light intensity of 400 mW/cm^2^, which is less by a factor of 8.5 than the intensity needed for homeotropic-aligned LC cells with the same composition. Even a battery-operated laser pointer induced the diffraction ring formation due to molecular reorientation. Furthermore, the film showed incident direction sensitivity, hence acting as a simple nonlinear optical diode. A large-area flexible film of the studied materials can be fabricated by display technology, which enables much wider applications of nonlinear optics.

## Methods

### Cell fabrication

Our material comprises a host of nematic LC 4-cyano-4’-pentylbiphenyl (5CB) and monoacrylate 4-[4-(4’-cyanobiphenyl)oxy]butyl acrylate (A4CB, A4CB:5CB = 10:90, molar ratio), doped with a photoactive guest dye molecule 5,5′′-bis-(5-butyl-2-thienylethynyl)-2,2′:5′,2′′-terthiophene (TR5, 0.1 mol% to the host) as well as the photoinitiator (Irgacure 651, 0.5 mol% to the host). The chemical structures of those compounds are provided in Supplementary Fig. S4. The mixture was inserted into a 100 μm thick hybrid glass cell, in which one surface was treated with silane coupler while the other was rubbed. This provided the hybrid alignment. Finally, photopolymerization was performed with 366 nm UV light followed by an annealing process.

## Author Contributions

A.S. and J.W. conceived and designed the study. J.W. performed the experiments. J.W. and A.S. analyzed the data. Y.A. and J.M. helped with the chemical syntheses and sample preparation. Y.A. and M.K. helped to design the optical setup and provided technical support for the optical experiments. J.W., A.S., and A.P. wrote the manuscript. All authors reviewed the final version of the manuscript.

## Additional Information

**How to cite this article**: Wang, J. *et al.* Laser-pointer-induced self-focusing effect in hybrid-aligned dye-doped liquid crystals. *Sci. Rep.* **5**, 09890; doi: 10.1038/srep09890 (2015).

## Supplementary Material

Supplementary Information

Supplementary Movie 1

Supplementary Movie 2
