# Supplementary material for: Laser-pointer-induced self-focusing effect in hybrid-aligned dye-doped liquid crystals
Source: Sci Rep. 2015 May 6;5:9890. doi: 10.1038/srep09890 (PMC4421798; doi:10.1038/srep09890)
Supplement: Supplementary Information [file srep09890-s1.doc]

**Supplementary information**

**Laser-pointer-induced self-focusing effect in hybrid-aligned dye-doped liquid crystals**

Jing Wang^1^, Yosuke Aihara^1^, Motoi Kinoshita^1,2^, Jun-ichi Mamiya^1^, Arri Priimagi^3^ & Atsushi Shishido^1,4^*

1. Chemical Resources Laboratory, Tokyo Institute of Technology, R1-12, 4259 Nagatsuta, Midori-ku, Yokohama 226-8503, Japan
2. Current address: Saitama Institute of Technology, 1690 Fusaiji, Fukaya, Saitama 369-0293, Japan.
3. Department of Chemistry and Bioengineering, Tampere University of Technology, Korkeakoulunkatu 8, 33720 Tampere, Finland
4. PRESTO, JST, 4-1-8 Honcho, Kawaguchi 332-0012, Japan

*e-mail: ashishid@res.titech.ac.jp

**1. Probe beam experiment**

A probe beam experiment was conducted to determine if the rings formed were generated by the photothermal effect or order-to-order molecular reorientation, by observing the polarization dependence of the ring pattern. Besides the Ar^+^ laser beam, a non-resonant He-Ne probe beam was applied at the same time and onto the same spot of the cell (Supplementary Fig. S1a). A colour filter was placed behind the cell to cut the Ar^+^ beam so that we could monitor the He-Ne beam while changing its polarization. For homeotropic-90, the rings exhibited no light polarization dependence (Supplementary Fig. S1c), suggesting that the molecules experienced an order-to-disorder transition. Hence, the rings were caused by the photothermal effect. On the other hand, rings in the arrangements homogeneous-0 and homeotropic-0 exhibited clear polarization dependence (Supplementary Fig. S1b and Fig. S1d), which indicated that, in these cases, the ring formation could be attributed to photoinduced order-to-order molecular reorientation.

**
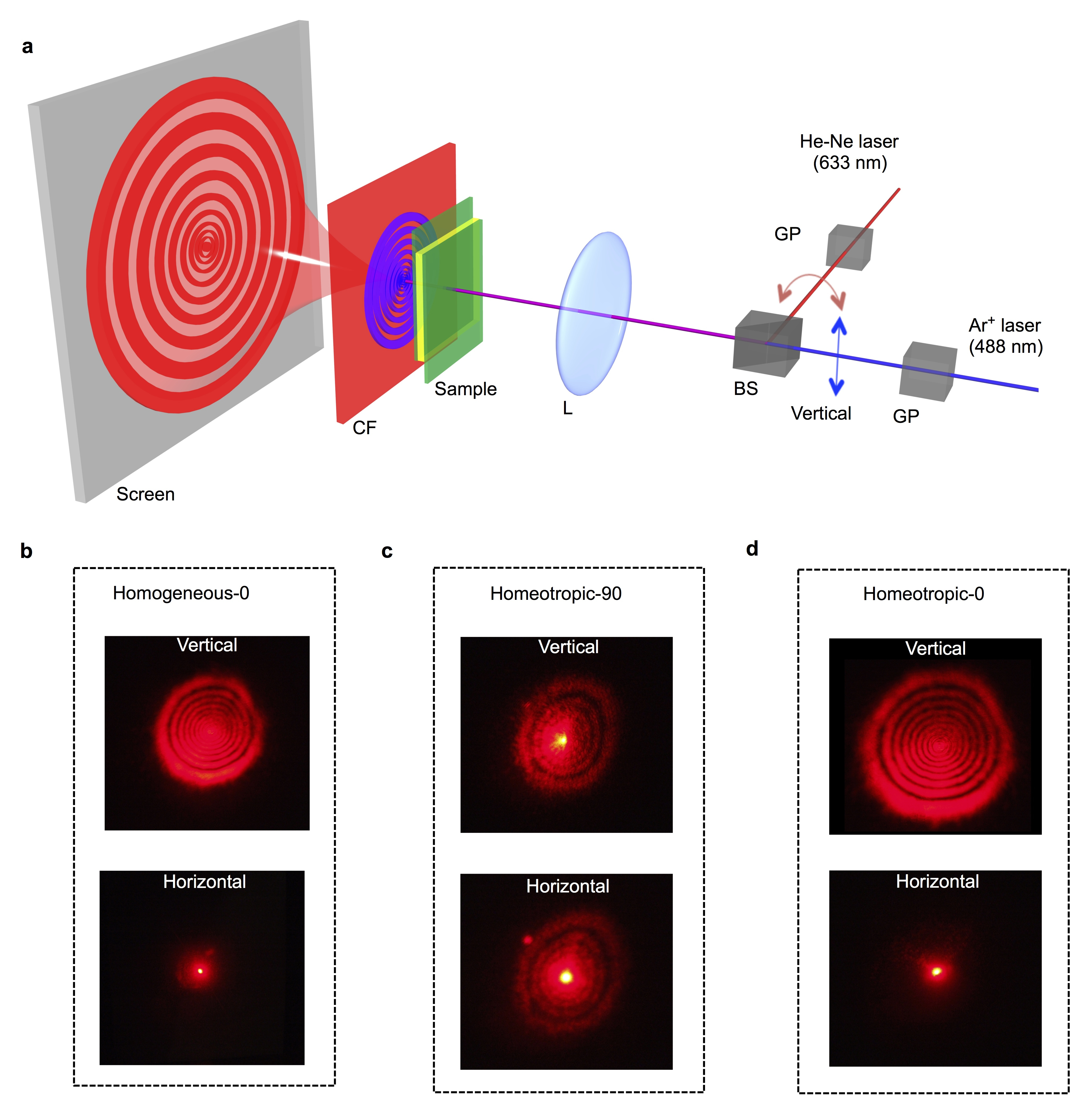
**

**Supplementary Figure S1. Probe beam method to determine the cause of the formed diffraction ring pattern.** (a) Optical setup. Ring pattern polarization dependence at cell arrangement of (b) homogeneous-0, (c) homeotropic-90, (d) homeotropic-0.

**2. Transmittance measurements for reversibility and response time evaluation**

Optical reversibility and response time was determined by measuring the transmittance change at the centre of the rings. As shown in Supplementary Fig. S2a, a non-resonant He-Ne probe beam was incident to the same spot on the cell as the Ar^+^ laser beam. After the Ar^+^ laser beam was cut by a colour filter, an iris was placed so that only the centre of the He-Ne beam ring pattern could go though the iris. Then, we recorded the transmittance change of the centre of the rings by a photodetector. The reversibility of the nonlinear response in a hybrid-aligned cell was confirmed by repeatedly measuring the transmittance change upon five subsequent on-off cycles with 200 s intervals (Supplementary Fig. S2b). Also, the optical response in the hybrid-aligned cell was accelerated by a factor of 10 compared to that of the homeotropic-aligned cell (Supplementary Fig. S2c).


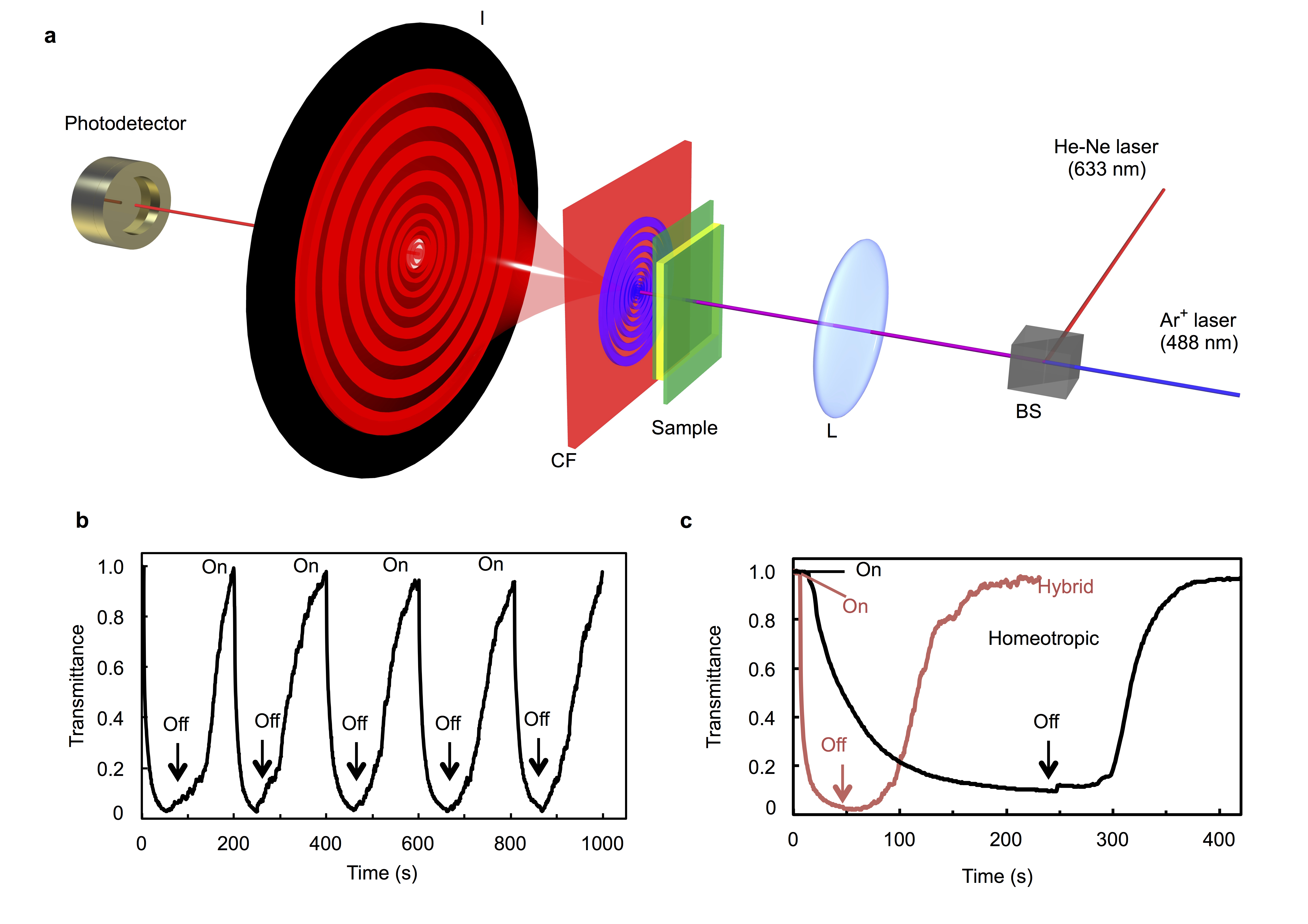


**Supplementary Figure S2. Optical reversibility and response time evaluation by transmittance measurement.** (a) Optical setup for transmittance measurement. L, lens; BS, beam splitter; CF, colour filter; I, Iris. (b) Reversibility in hybrid-aligned TR5-doped PSLC. (c) Comparison of response time between hybrid-aligned and homeotropic-aligned TR5-doped PSLC.

**3. UV-Vis absorption spectra**

Polarized absorption spectra were measured for hybrid-aligned PSLC (0.10 and 0.03 mol% TR5) and hometropic-aligned PSLC (0.10 and 0.32 mol% TR5), as shown in Supplementary Fig. 3. For the hybrid-aligned cells, four lines show the spectra at four different cell arrangements based on the surface facing the light and the angle between the rubbing direction and polarization. As shown in Supplementary Fig. S3a, the hybrid-aligned TR5 (0.10 mol%)-doped PSLC had similar absorbance at homeotropic-90 and homogeneous-90, with much higher absorbance at hometropic-0 and highest absorbance at homogeneous-0. Supplemetary Fig. S3b and S3c show the spectra measured with two orthogonal polarizations for the homeotropic-aligned cells. At the wavelength of 488 nm (Ar^+^ laser), the absorbances for hybrid-aligned PSLC (0.10 mol% TR5, homeotropic-0) and homeotropic-aligned PSLC (0.10 and 0.32 mol% TR5) films are 0.45, 0.14 and 0.44, respectively. At a wavelength of 460 nm (laser pointer), the absorbance for the hybrid-aligned TR5 (0.03 mol%)-doped PSLC film was 0.39, which was close to that of the hybrid-aligned (0.10 mol% TR5) film at 488 nm.


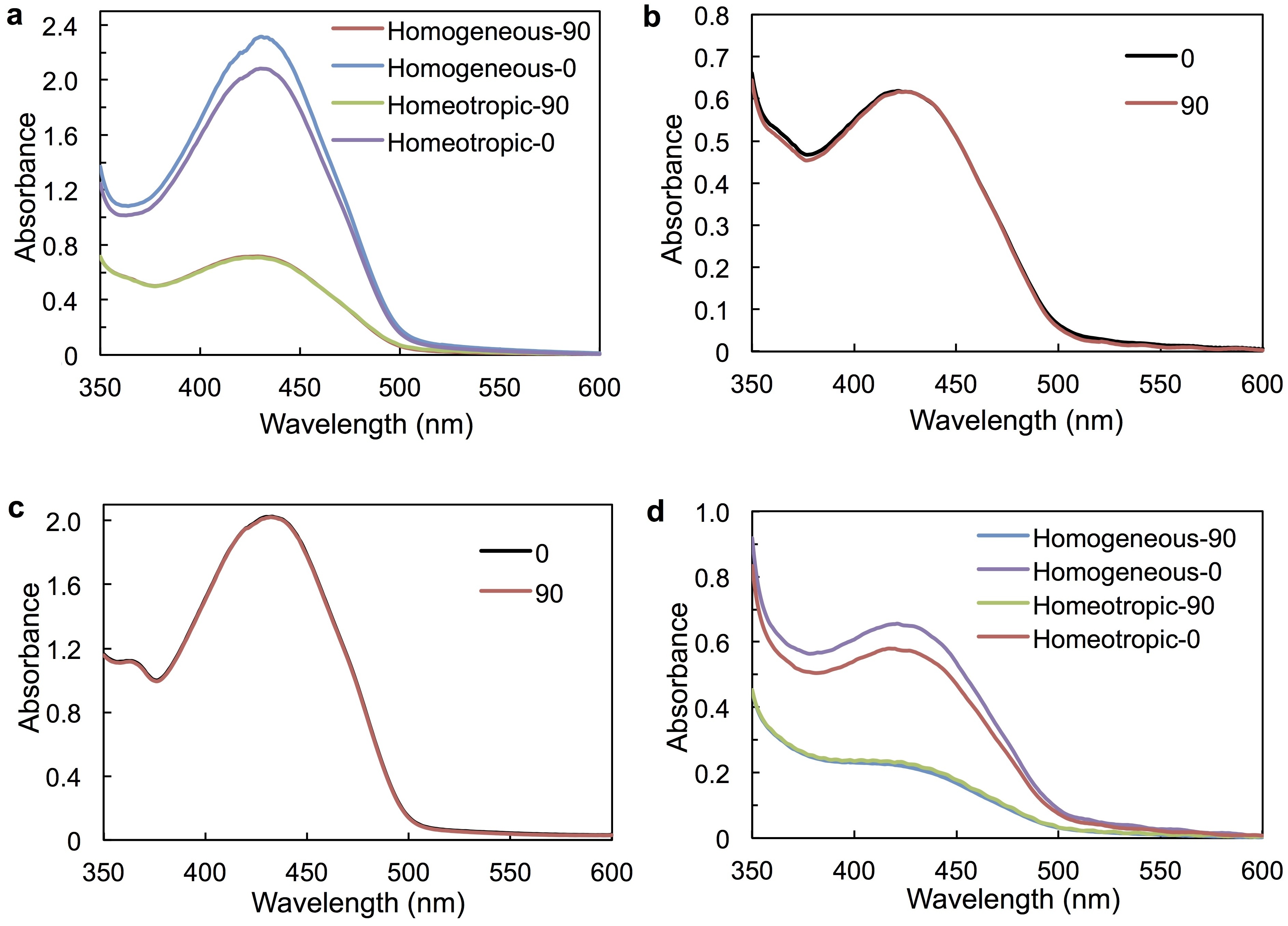


**Supplementary Figure S3. Polarized absorption spectra** **of** (a) hybrid-aligned PSLC (0.1 mol% TR5) at four cell arrangements, (b) homeotropic-aligned PSLC (0.1 mol% TR5), (c) homeotropic-aligned PSLC (0.32 mol% TR5), (d) hybrid-aligned PSLC (0.03 mol% TR5) at four cell arrangements.

**4. Chemical Structures**

5,5′′-bis-(5-butyl-2-thienylethynyl)-2,2′:5′,2′′-terthiophene (TR5) was synthesized following reported methods^1^ and used as the photoactive guest dye molecule. 4-cyano-4’-pentylbiphenyl (5CB, Merck Ltd.) served as the host LC, which has a nematic phase at room temperature. 4-[4-(4’-cyanobiphenyl)oxy]butyl acrylate (A4CB) was synthesized and used as the photopolymerizable monoacrylate to prepare polymer-stabilized LCs. A4CB has a similar structure as 5CB, which made it soluble in the main host LC. Irgacure 651 was chosen as the photoinitiator for photopolymerization.


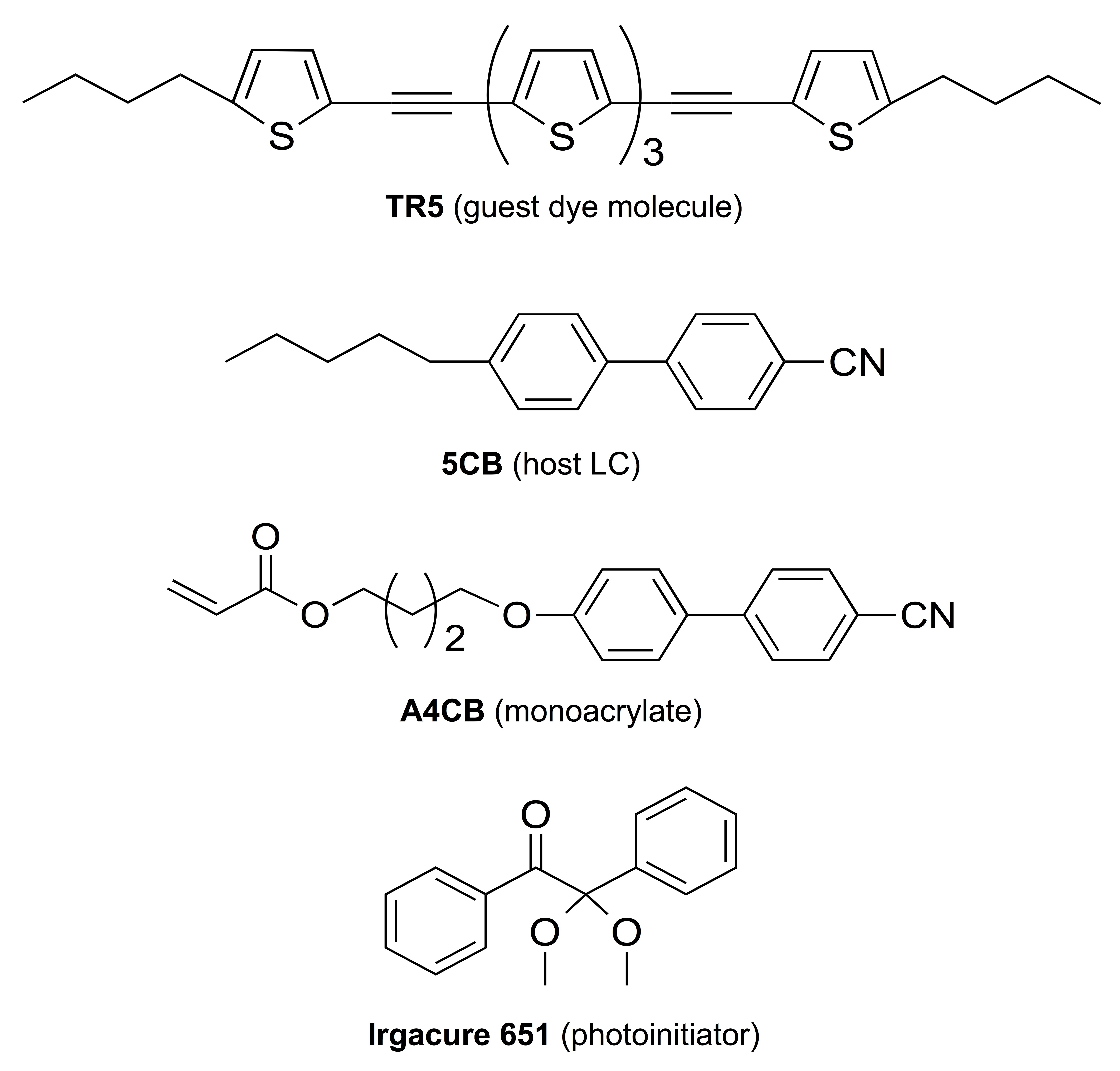


**Supplementary Figure S4.** Chemical structure of the compounds used in this study.

**5. Laser-pointer-driven self-focusing effect**

**Supplementary Movie S1.** Movie of the demonstration of laser-pointer (466 nm, 1 mW)-driven self-focusing effect in hybrid-aligned TR5-doped PSLC film.

**6. Bifacial optical property**

**Supplementary Movie S2.** Movie of the demonstration of the optical bifacial property in hybrid-aligned TR5-doped PSLC film using a laser pointer (466 nm, 1 mW).

**Reference**

1. Zhang, H. *et al*. A thiophene liquid crystal as a novel π-conjugated dye for photo-manipulation of molecular alignment. *Adv. Mater*. **12**, 1336-1339 (2000).
